# *Schisandra rubriflora* Plant Material and In Vitro Microshoot Cultures as Rich Sources of Natural Phenolic Antioxidants

**DOI:** 10.3390/antiox9060488

**Published:** 2020-06-04

**Authors:** Agnieszka Szopa, Michał Dziurka, Sebastian Granica, Marta Klimek-Szczykutowicz, Paweł Kubica, Angelika Warzecha, Karolina Jafernik, Halina Ekiert

**Affiliations:** 1 Chair and Department of Pharmaceutical Botany, Jagiellonian University, Medical College, ul. Medyczna 9, 30-688 Kraków, Poland; marta.klimek-szczykutowicz@doctoral.uj.edu.pl (M.K.-S.); p.kubica@uj.edu.pl (P.K.); a.warzecha@student.uj.edu.pl (A.W.); karolina.jafernik@doctoral.uj.edu.pl (K.J.); halina.ekiert@uj.edu.pl (H.E.); 2 Polish Academy of Sciences, The Franciszek Górski Institute of Plant Physiology, ul. Niezapominajek 21, 30-239 Kraków, Poland; m.dziurka@ifr-pan.edu.pl; 3 Department of Pharmacognosy and Molecular Basis and Phytotherapy, Medical University of Warsaw, ul. Banacha 1, 02-097 Warszawa, Poland; sgranica@wum.edu.pl

**Keywords:** red-flowered Chinese magnolia vine, in vitro cultures, plant biotechnology, UHPLC-DAD-ESI-MS^3^, HPLC-DAD, ROS scavenging activity, phenolic acids, flavonoids, polyphenols, antioxidants

## Abstract

*Schisandra rubriflora* is a dioecious, underestimated medicinal plant species known from traditional Chinese medicine. The present study was aimed at characterising the polyphenolic profile composition and the related antioxidant capacity of *S. rubriflora* fruit, stem and leaf and in vitro microshoot culture extracts. Separate analyses of material from female and male specimens were carried out. This study was specifically aimed at detailed characterisation of the contribution of phenolic compounds to overall antioxidant activity using ultra-high-performance liquid chromatography with a photodiode array detector coupled to electrospray ionization ion trap mass spectrometry (UHPLC-DAD-ESI-MS^3^) and a high-performance liquid chromatography-diode array detector (HPLC-DAD). Using UHPLC-DAD-ESI-MS^3^, twenty-seven phenolic compounds from among phenolic acids and flavonoids were identified. Concentrations of three phenolic acids (neochlorogenic, chlorogenic and cryptochlorogenic acids) and eight flavonoids (hyperoside, rutoside, isoquercitrin, guaijaverin, trifolin, quercetin, kaempferol, and isorhamnetin) were determined using HPLC-DAD using reference standards. The highest total phenolic content was confirmed for the stem and leaf extracts collected in spring. The contents of phenolic compounds of in vitro biomasses were comparable to that in the fruit extracts. The methanolic extracts from the studied plant materials were evaluated for their antioxidant properties using various in vitro assays, namely free radicals scavenging estimation using 2,2-diphenyl-1-picryl-hydrazyl-hydrate (DPPH), ferric-reducing antioxidant power (FRAP) and cupric-reducing antioxidant capacity (CUPRAC) as well as QUick, Easy, New, CHEap, and Reproducible CUPRAC (QUENCHER-CUPRAC) assays. A close relationship between the content of polyphenolic compounds in *S. rubriflora* and their antioxidant potential has been documented.

## 1. Introduction

Currently, the attention of scientific research has been focused on plants as a source of phytochemicals with antioxidant potential. Different groups of plant secondary metabolites have been identified as responsible for this activity. These natural compounds comprise different structures and involve several protective mechanisms. Plant secondary metabolites most likely to exhibit health-promoting effects include polyphenols such as flavonoids, phenolic acids, catechins, tannins, and proanthocyanidins [1,2,3,4,5,6]. Several studies reported that these compounds show a preventive effect, associated with an excess of free radicals, e.g., against cancer, atherosclerosis, Alzheimer’s disease, Parkinson’s disease, and ischemic and cardiovascular diseases, etc. [7,8]. Furthermore, the latest studies of plant biotechnology proved that different in vitro systems of various plant species could be a rich, alternative source of polyphenolic compounds of strong antioxidant power, even higher than that of the intact plants [9,10,11].

*Schisandra chinensis* (Turcz.) Baill is a well-known plant species, used in the traditional Chinese medicine, whose importance is increasing nowadays in European and American countries as well [12,13,14,15,16]. Its fruits are a widely-consumed nutraceutical providing beneficial nutritional and bioactive properties [17,18]. *S. chinensis* fruits are known for their hepatoprotective, anticancer, antiaging, and stimulant effects as well as use as a sedative and tonic drug [12,17]. In the *Schisandra* genus, the most recognised active components are lignans (especially from dibenzocyclooctadiene group) including, among others, schisandrin, gomisins A, C and G, deoxyschisandrin, schisanhenol, and schisantherins A and B [12,19,20]. This plant has been frequently studied for its antioxidant potential [21,22,23,24]. The results of the scientific studies have shown that lignans are not the main components responsible for the antioxidant activity. The polyphenolic fraction of the studied extracts have been indicated as responsible for this activity [24,25,26,27,28,29]. This is a very interesting aspect, as there is little research on *S. chinensis* polyphenolic composition [23,24,30].

*Schisandra rubriflora* (Franch.) Rehd. et Wils is another species of the genus *Schisandra* known from East Asian phytotherapy. The species seems to be closely related to *S. chinensis* but less known, as it is the endemic species (typical for the Sichuan province of China) [31,32]. The planting of *S. rubriflora* outside the East Asian region is limited [14,32]. *S. rubriflora* is a dioecious vine, whose fruits are known for their use in traditional Chinese medicine as sedatives and toning agents. This species is also traditionally used in the treatment of hepatitis, chronic gastroenteritis and neurasthenia [32,33]. To date, the potential pharmacological applications of *S. rubriflora* fruit been described only by the Chinese research groups. AntiHIV-1 studies (inhibition of HIV-1 replication in H9 lymphocytes) were performed for fruit extracts [34,35]. Shoot extracts have been shown to be useful in the treatment of liver and bile duct disorders, through studies assessing their impact on the level of glutamin-pyruvate transaminase (GPT) in blood [32,36]. According to these studies, compounds from dibenzocyclooctadiene lignans as well as triterpenoids are responsible for these properties [34,37,38]. Recently, we have studied the lignan profiling of fruits, leaves and stems of female (F) and male (M) *S. rubriflora* plants [39]. In the same study, strong antiinflammatory properties in this species were also noted, based on inhibitory activity against 15-lipooxygenase (15-LOX), phospholipase A_2_ (sPLA_2_), cyclooxygenase 1 and 2 (COX-1; COX-2) enzyme assays have been indicated [39].

The aim of this study was to investigate the polyphenol profile and the antioxidant activity of *S. rubriflora* to fill this gap in the literature. The present study has presented the phytochemical qualitative and quantitative characteristics of the polyphenol content of *S. rubriflora* dividing the material into female (F) and male (M) specimens for the first time. For the estimations an ultra-high-performance liquid chromatography with a photodiode array detector coupled to electrospray ionization ion trap mass spectrometry (UHPLC-DAD-ESI-MS^3^) and a high-performance liquid chromatography-diode array detector (HPLC-DAD) were used. Fruits, stems and leaves of the soil-grown plants as well F and M lines of *S. rubriflora* in vitro microshoot cultures were studied. Moreover, total phenolic content was measured with Folin-Ciocalteu reagent. The antioxidant potential in the tested plant materials has been investigated for the first time using different methods, namely scavenging of free radicals using 2,2-Diphenyl-1-Picryl-Hydrazyl-Hydrate (DPPH), Ferric-Reducing Antioxidant Power (FRAP), Cupric-Reducing Antioxidant Capacity (CUPRAC) as well as QUick, Easy, New, CHEap, and Reproducible CUPRAC (QUENCHER-CUPRAC).

## 2. Materials and Methods

### 2.1. Reagents

Acetic acid, ethanol 96%, methanol, and sucrose were from Chempur (Piekary Śląskie, Poland). HPLC-grade methanol and acetonitrile were purchased in Merck (Darmstadt, Germany).

Plant culture media components, plant growth regulators BA (6-benzyladenine) and NAA (1-naphthaleneacetic acid) and agar were purchased in Duchefa Biochemie (Haarlem, Netherlands). Cultures were grown in the plant tissue-dedicated glass containers (V8630, Sigma-Aldrich, Saint Louis, MI, USA).

Commercially available standards: chlorogenic acid, cryptochlorogenic acid, and neochlorogenic acid, hyperoside (quercetin 3-galactoside), isoquercitrin (quercetin 3-glucoside), isorhamnetin, kaempferol, guaijaverin (quercetin 3-arabinoside), quercetin, rutoside (quercetin 3-rutinoside), and trifolin (kaempferol-3-galactoside) of HPLC grade (≥95.0%) purity were acquired in Sigma-Aldrich Saint Louis, MI, USA. Ammonium acetate, CuCl_2_⋅2H_2_O, DPPH, FeCl_3_⋅6H_2_O, Folin-Ciocalteu reagent, hydrochloric acid (HCl), Na_2_CO_3_, 2,9-dimethyl-1,10-phenanthroline (neocuprine), 2,4,6-tris(2-pyridyl)-s-triazine (TPTZ), and (±)-6-hydroxy-2,5,7,8-tetramethylchromane-2-carboxylic acid (trolox) were also provided by Sigma-Aldrich. Deionised water (>15 MΩ) was produced in house (PureLab OptionR, Elga, High Wycombe, UK).

### 2.2. Plant Material

Plant material for establishing in vitro cultures, as well as stems, leaves and fruits of the intact plants, were obtained from Clematis—the Source of Good Climbers Ltd. (Źródło Dobrych Pnączy Spółka z o.o., Pruszków, Poland) [40]. The plant species were identified by PhD Szczepan Marczyński (the head of the Clematis arboretum). Plant material was harvested in 2018 during three vegetative periods from 10-year-old female (F) and male (M) individuals of *Schisandra rubriflora* (Franch.) Rehd. et Wils specimens. Leaves and stems (stems were collected with leaves as they grow) were harvested in spring (May), summer (July) and autumn (September) from F and M specimens (approximately 50 individuals each). Fruits were collected in September 2018 from F specimens.

Leaf buds which were used for the initiation of in vitro cultures were collected in April 2018. Fruits were lyophilised and leaves and stems were air dried (at 25–30 °C). The dry plant material was pulverised in a mixing mill (MM 400, Retch, Germany).

### 2.3. Establishment of In Vitro Cultures

*S. rubriflora* leaf buds of F and M specimens were degreased with 70% ethanol (30 s) and then subjected to further sterilisation steps. HgCl_2_ (mercury chloride II) at a concentration of 0.1% was used for sterilisation for 7 min. Sterile buds were rinsed with sterile redistilled water and transferred to the agar medium according to Murashige and Skoog (1962) (MS) [41] and supplemented with the following plant growth regulators (PGRs): 1 mg/L 6-benzyladenine (BA) and 0.5 mg/L 1-naphthaleneacetic acid (NAA). Microshoots appeared after four weeks, and then (after eight weeks) stable in vitro cultures were obtained. Cultures were subcultured every 30 days.

### 2.4. Experimental Microshoot Cultures

Experimental agar microshoot cultures were maintained on MS medium [41] with 0.72% (*w*/*v*) agar and 3% (*w*/*v*) sucrose. In vitro cultures were cultured at 25 ± 2 °C under continuous artificial illumination of LED white light, with the photosynthetic photon flux density (PPFD) of 40 μmol m^−2^ s^−1^, and subcultured at 30 day intervals.

For the experiment, 0.5 g of inoculum (initial fresh weight of microshoots) per vessel was used. For experimental microshoot cultures under optimisation of PGR composition performed before [unpublished], the best medium for their cultivation contained 1 mg/L BA and 1 mg/L indole-3-butyric acid (IBA). The duration of the growth cycle time was 30 days; three series (*n* = 10) were performed.

### 2.5. UHPLC-DAD-ESI-MS^3^

Extracts from female (F) and male (M) individuals of *S. rubriflora* were prepared through sonication (15 min, room temperature) of 100 mg of the dry plant material with 2 mL of methanol:water (1:1, *v*/*v*). The samples were centrifuged and the supernatant was filtrated through a 0.45 μm polyvinylidene fluoride (PVDF) syringe filter before UHPLC analysis.

UHPLC-DAD-ESI-MS_3_ was performed using the Ultimate 3000 series system (Dionex, Idstein, Germany) coupled with an amaZon SL ion trap mass spectrometer (Bruker Daltonik GmbH, Bremen, Germany). The analysis of compounds was carried out using a Kinetex XB-C^18^ analytical column (150 mm × 2.1 mm × 1.9 µm), Phenomenex (Torrance, CA, USA). The column temperature was 25 °C. Elution was conducted using a mobile phase A (0.1% methanol in water) and a mobile phase B (0.1% methanol in acetonitrile) with a three-step gradient: 0 min 4% B, 60 min 26% B and 90 min 95% B. The flow rate was 0.300 mL/min during all the analyses. A volume of 3 µL of the prepared extract was injected to the column. UV-Vis spectra were recorded in the range of 200–450 nm. The chromatogram was read at 254 nm. The eluate was introduced into the mass spectrometer without splitting. The amaZon SL ion trap mass spectrometer was equipped with an ESI source. The parameters for the source were set as follows: nebuliser pressure 40 psi; dry gas flow 9 L/min; dry temperature 145 °C; and capillary voltage 4500 V. The analysis was carried in a scan range of 70–2200 *m/z*. Compounds were analysed in the negative and positive ion modes. MS^2^ and MS^3^ fragmentations were performed using the Smart Frag mode. Compounds were tentatively identified by determination of their molecular mass, UV-Vis spectra and fragmentation profiles in respect to the literature data and by comparison to available standards.

### 2.6. HPLC-DAD 

After conducting UHPLC-DAD-ESI-MS^3^ for quantification of the detected compounds, HPLC-DAD was performed.

In order to prepare the methanolic extracts, 0.3 g samples of the dry powder in vitro biomass and in vivo-derived plant material were weighed (three samples in three replications). The material was subjected to extraction with HPLC-grade methanol (5 mL). The extraction was carried out in an ultrasonic bath (Sonic-2, POLSONIC, Warsaw, Poland; ultrasonic power 2 × 100 W, 40 kHz, 1.6 L volume) twice for 30 min at 25 ± 2 °C. The extracts were centrifuged (MPW-223E, MPW, Warsaw, Poland) at 4000 rpm for 5 min. The extracts were then filtered through the syringe filters (0.22 µm Millex^®^GP, Millipore, Merck, Darmstadt, Germany).

Quantitative analysis of phenolic compounds in the methanolic extracts was performed according to a validated method [42,43], using Merck-Hitachi liquid chromatograph (LaChrom Elite, Darmstadt, Germany) with a DAD L-2455 detector. The separation was conducted on a Purospher RP-18 (250 × 4 mm; 5 μm, Merck, Germany) column. The mobile phase consisted of A—methanol, 0.5% acetic acid 1:4; B—methanol (*v*/*v*). The flow rate was 1 mL/min at 25 °C. The gradient was as follows: 100% A for 0–20 min; 100–80% A for 20–35 min; 80–70% A and 20–30% B for 35–45 min; 70–60% A and 30–40% B for 45–55 min; 60–50% A and 40–50% B for 55–60 min; 50–25% A and 50–75% B for 60–65 min; 25–0% A and 75–100% B for 65–70 min; 0–0% A and 100–100% B for 70–75 min; 0–100% A and 100–0% B for 75–80 min; 100–100% A and 0–0% B for 80–90 min. The injection volume was 10 μL and the compounds were detected at 254 nm. Identification and quantification were performed by comparison to retention times of parameters and confirmed later by fragmentation spectra (UHPLC-DAD-ESI-MS^3^). Quantification was performed based on calibration curves. The results were expressed in mg/100 g dry weight (DW).

### 2.7. The Total Phenolic Assay

Total phenolic content was measured for microshoot cultures, fruits and plant material (stems and leaves) collected in spring, with the highest phenolic content determined by HPLC-DAD.

Approximately 3 mg of the weighted samples was extracted in 1 mL of analytical-grade methanol (5 min, 15 Hz, MM400 RetschHaan, Germany), and then centrifuged (3 min, 15 °C, 33,000× *g*, 32R, Hettich, Balingen, Germany). Total phenolic content was measured according to the method by Singelton et al. [44] with modifications reported by Bach et al. [45]. A volume of 100 μL of extract was mixed with 0.45 mL of Folin-Ciocalteu reagent in deionised water (5/2 *v*/*v*), and then 0.45 mL of saturated Na_2_CO_3_ was added. Samples were incubated in the dark (2 h, 25 °C), and then centrifuged and transferred to well plates. The absorbance was measured at 760 nm. The analyses were carried out in a 96-well plate using Synergy II (Biotek, Winooski, VT, USA) reader. The antioxidant response was expressed as mg trolox equivalents (TE)/100 g of dry weight (DW).

### 2.8. The Antioxidant Potential Estimation

The extract collected for the total phenolic assay was used to carry out the antioxidant assays. All measurements were performed in a 96-well plate using Synergy II (Biotek, Winooski, VT, USA) reader. The antioxidant response was expressed as TE in mg/100 g DW. All measurements were performed in five replicates.

#### 2.8.1. The FRAP Assay

The ferric-reducing ability of the extracts was assessed using the FRAP assay [46]. A solution of TPTZ (10 mmol/L in 40 mmol/L HCl) was mixed with 20 mmol/L FeCl_3_⋅6H_2_O and 300 mmol/L acetate buffer at pH = 3.6 (1/1/10 *v*/*v*/*v*). A volume of 50 μL of the studied extract was added to 150 μL of this mixture. The samples were incubated for 5 min at 25 °C. The absorbance was read at 593 nm.

#### 2.8.2. The DPPH Assay

Free radical-scavenging activity was estimated using DPPH [47]. Plant extract (50 μL) was mixed with 150 μL of the DPPH methanolic solution. After incubation on a horizontal shaker in the dark (1 h, 25 °C), the absorbance was read at 517 nm.

#### 2.8.3. The CUPRAC Assay of the Total Antioxidant Capacity

The cupric-reducing antioxidant capacity (CUPRAC) assay [48] adapted by Biesaga-Kościelniak et al. [49] was used. A volume of 50 μL of 1 mol/L ammonia acetate buffer (pH = 7) and 50 μL of 7.5 mmol neocuprine in methanol were mixed with 50 μL of sample extract followed by 50 μL of 10 mmol Cu^2+^. After incubation on a horizontal shaker (15 min, 25 °C), the absorbance was read at 450 nm.

#### 2.8.4. The QUENCHER-CUPRAC Assay of the Total Antioxidant Capacity

The QUENCHER-CUPRAC assay [50] was used for estimation of the aggregate antioxidant response of plant material. The 1 mL aliquots of 1 mol/L ammonia acetate buffer (pH = 7.0), 7.5 mmol/L neocuprine, and 10 mmol/L Cu^2+^ were added sequentially to 1 mg of accurately weighed pulverised samples. After a 2 h incubation (shaking on a rotator, 25 °C), the samples were centrifuged and transferred to 96-well plates. The absorbance was measured at λ = 425 nm.

### 2.9. The Statistical Analysis

The quantitative HPLC results were expressed in mg/100 g dry weight (DW). The total phenolic and antioxidant assays of trolox equivalents (TE) in mg/100 g DW. The results were expressed as the mean ± standard deviation (SD) of four or five samples (*n* = 4, *n* = 5, *p* < 0.05) for experiments that were repeated three times.

## 3. Results

### 3.1. The Characteristics of In Vitro Cultures

**Figure 1:**
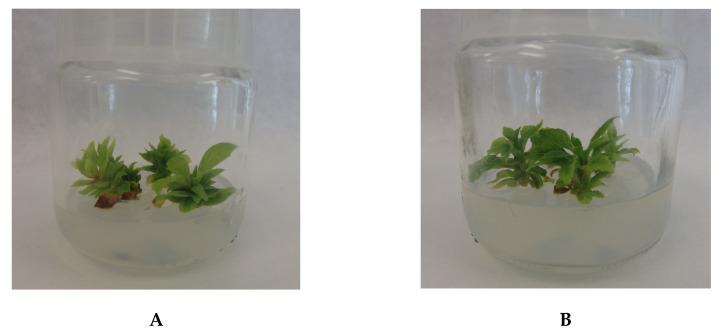
The morphological appearance of *Schisandra rubriflora* microshoots from agar in vitro cultures (MS medium with 1 mg/L 6-benzyladenine (BA) and 0.5 mg/L 1-naphthaleneacetic acid (NAA), after a 30 day growth period): (**A**) female (F) line; (**B**) male (M) line.

The morphological differences in the appearance of microshoot cultures of F and M culture lines grown on MS medium variant with 1 mg/L BA and 1 mg/L IBA were not measurable (Figure 1). It was possible to observe a significant number of dark green, strongly spread microshoots. The dry biomass increments were expressed by the growth index (Gi) (Gi = (Dw_1_ − Dw_0_)/Dw_0_, where Dw_1_—dry weight of microshoots at the end of the experiment; Dw_0_—dry weight of the inoculum) [51]. After a 30 day growth period, Gi was 2.91 ± 0.28 and 3.22 ± 0.31 for the F and M lines, respectively.

### 3.2. UHPLC-DAD-ESI-MS^3^ Analysis of Phenolics

The qualitative profiles of phenolic compounds from in vitro biomass extracts as well as from the intact plants (stems and leaves) were the same. In the fruit extracts, neochlorogenic acid was not detected.

**Figure 2:**
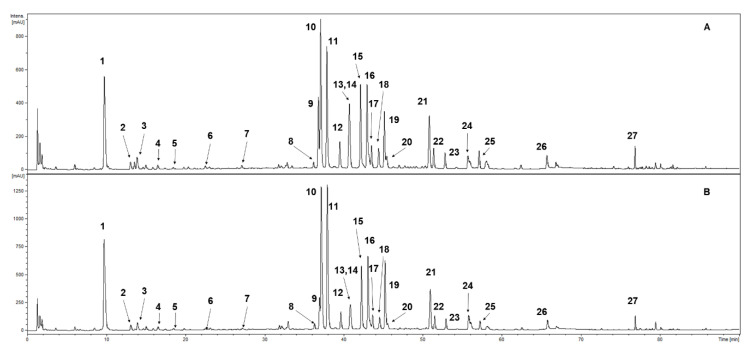
UHPLC-DAD-ESI-MS^3^ chromatogram of *S. rubriflora* leaf extract separation of the phenolic compounds (at 254 nm): (**A**) female (F) specimen; (**B**) male (M) specimen. For compound numbers, refer to Table 1.

UHPLC-DAD-ESI-MS^3^ of the methanolic extracts confirmed the presence of 27 phenolic compounds (Table 1, Figure 1). Compounds were classified into photochemical groups based on the profile of UV-Vis spectra recorded during the analysis. Constituents with an absorption maxima in UV at ca. 300 and ca. 325 nm were classed as caffeic acid derivatives (1, 4 and 5). The characteristic maxima at ca. 300 and ca. 310 nm were tentatively identified as p-coumaroyl acid derivatives (2, 3, 6, and 7). Compounds with a strong absorption maxima at ca. 340–360 nm were classed as flavonoid derivatives. Further characterisation was performed using analysis of MS and MS/MS spectra. Compounds with an aglycone signal in MS^2^ or MS^3^ spectra at *m/z* = 301 in the negative ion mode were identified as quercetin derivatives (8–12, 14, and 19). Compounds exhibiting a strong signal in MS/MS spectra at *m/z* = 285 in the negative ion mode were classed as kaempferol glycosides (13, 15, 16, 18, 20, 21, and 25) with various substitution patterns. Constituents an aglycone signal in the fragmentation spectra at *m/z* = 315 in the negative ion mode were identified as isorhamentin derivatives (17 and 22). Three free aglycones were detected and identified as quercetin (24), kaempferol (26) and isorhamnetin (27). As a result of the comparison to the chemical standards, some of the detected natural products were fully identified as neochlorogenic acid (1), chlorogenic acid (4), cryptochlorogenic acid (5), hyperoside (quercetin 3-galactoside) (9), rutoside (quercetin 3-rutinoside) (10), isoquercitrin (quercetin 3-glucoside) (11), guaijaverin (quercetin 3-arabinoside) (12), trifolin (kaempferol 3-*O*-galactoside) (13), avicularin (quercetin 3-*O*-α-L-arabinopyranoside) (14), and astragalin (kaempferol 3-glucoside) (16) (the numbers correspond with Figure 2 and Table 1).

### 3.3. HPLC-DAD Phenolic Profile Quantitative Characterisation

#### 3.3.1. Fruits

From among phenolic acids present in the studied fruit extracts of *S. rubriflora*, quantification was performed for chlorogenic acid and cryptochlorogenic acid. The presence of neochlorogenic acid was not detected. Total phenolic content was 78 mg/100 g DW (Table 2). Cryptochlorogenic acid was the dominant compound (69 mg/100 g DW). The amount of chlorogenic acid was lower (9 mg/100 g DW) (Table 2).

Five flavonoid glycosides—hyperoside, rutoside, isoquercitrin, guaijaverin, and trifolin—and three aglycones—quercetin, kaempferol and isorhamnetin—were quantitatively estimated in the fruit extracts (Table 2). The total content of flavonoids amounted to 353 mg/100 g DW. The dominant compounds were kaempferol (139 mg/100 g DW), guaijaverin (52 mg/100 g DW), rutoside (42 mg/100 g DW), and isorhamnetin (39 mg/100 g DW). The content of other compounds was as follows: quercetin (32 mg/100 g DW), isoquercitrin (27 mg/100 g DW) and trifolin (19 mg/100 g DW); the lowest content was found for hyperoside (42 mg/100 g DW) (Table 2).

#### 3.3.2. Stems

The individual and total amounts of the studied phenolic acids and flavonoids were dependent on the stem harvesting vegetation period and on the sex of *S. rubriflora* individuals.

The total phenolic acid content in the stem extracts ranged from 260 mg/100 g DW (M, summer) to 590 mg/100 g DW (F, spring) (Table 3). The quantitative estimations in the stem extracts were performed for neochlorogenic acid, chlorogenic acid and cryptochlorogenic acid. Neochlorogenic acid was the quantitatively dominant compound both in female (F) and male (M) stems collected during the vegetative season (Table 3). The amounts of this compound ranged from 185 mg/100 g DW (M, summer) to 457 mg/100 g DW (F, spring). The amounts of cryptochlorogenic and chlorogenic acids were also high, ranging from 20 to 80 mg/100 g DW and from 18 to 53 mg/100 g DW, respectively. The minimal and maximal contents of cryptochlorogenic and chlorogenic acids was confirmed in the same samples collected from F specimens in spring and in autumn, respectively. 

Five flavonoid glycosides—hyperoside, rutoside, isoquercitrin, guaijaverin, and trifolin—and three aglycones—quercetin, kaempferol and isorhamnetin—were quantitatively estimated in the stem extracts (Table 3). The total flavonoid content in the stem extracts varied from 967 (M, autumn) to 2693 mg/100 g DW (F, spring). Rutoside, isoquercitrin and trifolin were the main compounds both in female (F) and male (M) stems collected during the vegetative season (Table 3). The amounts of these compounds ranged from 213 (M, summer) to 932 mg/100 g DW (F, spring), 160 (M, autumn) to 624 mg/100 g DW (F, spring) and from 189 (M, autumn) to 605 mg/100 g DW (F, spring), respectively. The highest contents of quercetin (135 mg/100 g DW), kaempferol (113 mg/100 g DW) and isorhamnetin (53 mg/100 g DW) were detected in the extracts of stems collected in spring from F specimens. The maximal content of hyperoside (130 mg/100 g DW) was detected in stems harvested from M specimens in autumn, while the maximal content of guaijaverin (190 mg/100 g DW) was confirmed for stems harvested in spring, also from M specimens (Table 3).

#### 3.3.3. Leaves

The individual and total amounts of the researched phenolic acids and flavonoids were dependent on the leaf harvesting vegetation period and on the sex of the studied *S. rubriflora* individuals.

The total phenolic acid content in the leaf extracts ranged from 262 (F, summer) to 758 mg/100 g DW (F, autumn) (Table 4). In the stem extracts, the quantitative estimations were performed for neochlorogenic acid, chlorogenic acid and cryptochlorogenic acid. The estimations revealed that neochlorogenic acid was the dominant compound both in female (F) and male (M) stems collected during the vegetative season (Table 4). The amounts of this compound ranged from 217 (F, summer) to 607 mg/100 g DW (F, autumn). The amounts of cryptochlorogenic and chlorogenic acids were lower and they ranged from 19 (F, spring) to 94 mg/100 g DW (M, autumn) and from 17 DW (F, summer) to 93 mg/100 g DW (F, autumn), respectively. 

Five flavonoid glycosides—hyperoside, rutoside, isoquercitrin, guaijaverin, and trifolin—and three aglycones—quercetin, kaempferol and isorhamnetin—were quantitatively estimated in the leaf extracts (Table 4). The total flavonoid content in the stem extracts varied from 1248 (M, autumn) to 2838 mg/100 g DW (M, spring). Rutoside, isoquercitrin and trifolin were the dominant compounds both in female (F) and male (M) stems collected during the vegetative season (Table 4). The amounts of these compounds ranged from 311 (M, autumn) to 841 mg/100 g DW (M, spring), from 180 (M, autumn) to 503 mg/100 g DW (F, spring) and from 251 (M, autumn) to 714 mg/100 g DW (M, spring), respectively. The highest contents of guaijaverin (269 mg/100 g DW) and quercetin (213 mg/100 g DW) were detected in the extracts of leaves collected in spring from M specimens. The highest contents of hyperoside (164 mg/100 g DW) and isorhamnetin (59 mg/100 g DW) were detected in the extracts of leaves collected in autumn, also from M specimens. The highest content of kaempferol (111 mg/100 g DW) was detected in the extracts of leaves collected from F specimens in autumn (Table 4).

#### 3.3.4. In Vitro Cultures

In the studied *S. rubriflora* F and M line microshoot culture extracts, quantification was performed for neochlorogenic acid, chlorogenic acid and cryptochlorogenic acid from among phenolic acids. Total phenolic content was 188 (F) and 211 mg/100 g DW (M) (Table 5). The highest amounts were confirmed for neochlorogenic acid (F—81 mg/100 g DW; M—103 mg/100 g DW) and cryptochlorogenic acid (F—87 mg/100 g DW; M—91 mg/100 g DW). The amount of chlorogenic acid was lower (F—20 mg/100 g DW; M—16 mg/100 g DW).

Five flavonoid glycosides—hyperoside, rutoside, isoquercitrin, guaijaverin, and trifolin—and three aglycones—quercetin, kaempferol and isorhamnetin—were quantitatively estimated in the fruit extracts (Table 5). The total contents of flavonoids were 328 (F) and 420 mg/100 g DW (M). The dominant compounds were aglycones: quercetin (F—56 mg/100 g DW; M—87 mg/100 g DW), kaempferol (F—72 mg/100 g DW; M—91 mg/100 g DW) and isorhamnetin (F—72 mg/100 g DW; M—80 mg/100 g DW). From among glycosides, guaijaverin (F—38 mg/100 g DW; M—44 mg/100 g DW), rutoside (F—29 mg/100 g DW; M—42 mg/100 g DW) and trifolin (F—27 mg/100 g DW; M—37 mg/100 g DW) were detected in large quantities. The amounts of isoquercitrin (F—21 mg/100 g DW; M—26 mg/100 g DW) and hyperoside (F—14 mg/100 g DW; M—12 mg/100 g DW) were slightly lower (Table 5).

### 3.4. The Total Phenolic Assay

The total phenolic assay, performed in accordance with the Folin-Ciocalteu method, indicated the highest results for the leaf extracts (F—36,633 mg/100 g DW; M—31,207 mg/100 g DW) and the lowest for the fruit extracts (5181 mg/100 g DW) (Table 6). In the stem extracts, total phenolic content was 22,763 mg/100 g DW for F and 18,129 mg/100 g DW for M specimens. In vitro cultured microshoots contained 4729 mg/100 g DW for F and 6374 mg/100 g DW for M lines (Table 6).

### 3.5. The Antioxidant Potential Assays

#### 3.5.1. The FRAP Assay

The antioxidant potential, measured with the FRAP assay, was highest for the leaf extracts (F—19,720 mg/100 g DW; M—28,179 mg/100 g DW) (Table 7). Further, a high antioxidant potential was indicated for the fruit extracts—10,075 mg/100 g DW. The antioxidant content in the stem extracts estimated with the FRAP assay amounted to 7474 mg/100 g DW for F and 5893 mg/100 g DW for M specimens. The antioxidant potential for in vitro cultured microshoots was 2869 mg/100 g DW for F and 5178 mg/100 g DW for M lines (Table 7).

#### 3.5.2. The DPPH Assay

The antioxidant potential measured with DPPH indicated the highest results for the leaf and stem extracts: 8405 and 8363 mg/100 g DW and 7967 and 8340 mg/100 g DW for F and M specimens, respectively (Table 7). A lower antioxidant potential was indicated for the fruit extracts—2984 mg/100 g DW. The antioxidant potential measured with DPPH for in vitro cultured microshoots was 2964 mg/100 g DW for F and 3750 mg/100 g DW for M lines (Table 7).

#### 3.5.3. The CUPRAC Assay

The antioxidant potential measured with the CUPRAC assay showed the highest results for the leaf and stem extracts: 19,720 and 28,179 mg/100 g DW and 22,270 and 17,920 mg/100 g DW for F and M specimens, respectively (Table 7). A lower antioxidant potential was indicated for the fruit extracts—10,075 mg/100 g DW. The antioxidant potential measured with the CUPRAC assay for in vitro cultured microshoots was 2869 mg/100 g DW for F and 5178 mg/100 g DW for M lines (Table 7).

#### 3.5.4. The QUENCHER-CUPRAC Assay

The antioxidant potential measured with the QUENCHER-CUPRAC assay revealed the highest results for the leaf and stem extracts: 29,539 and 29,303 mg/100 g DW and 27,147 and 28,317 mg/100 g DW for F and M specimens, respectively (Table 7). A lower antioxidant potential was indicated for the fruit extracts (6083 mg/100 g DW). The QUENCHER-CUPRAC antioxidant potential measured for in vitro cultured microshoots was 5994 mg/100 g DW for F and 5822 mg/100 g DW for M lines (Table 7).

## 4. Discussion

In our study, the phenolic profile of *S. rubriflora* female (F) and male (M) specimens has been evaluated for the first time. This study divided specimens into F and M individuals as well as dividing different parts of plants and different vegetative periods of their harvesting. The research showed a relationship between the content of polyphenolic compounds in the extracts and their antioxidant potential, which was measured using four methods: DPPH, FRAP, CUPRAC and QUENCHER-CUPRAC. In addition, the F and M lines of *S. rubriflora* in vitro microshoot cultures were initiated and evaluated for phenolic content as well as antioxidant potential.

UHPLC-DAD-ESI-MS^3^ analyses provided the qualitative profile of *S. rubriflora* samples. The qualitative profiles of phenolic compounds from in vitro biomass extracts as well as from stems and leaves were the same. Neochlorogenic acid was the only absent compound in the fruit extracts. The analyses confirmed the presence of 27 phenolic compounds (Table 1, Figure 2). *S. rubriflora* has not been studied for phenolic compounds before. There is only a single study dealing with extracts from stems collected in Lincang County, China, in autumn. Li et al. [52], using various column chromatography methods (silica gel, Sephadex LH-20 and RP-18 high-resolution electrospray ionisation mass spectrometry (HRESIMS)) detected only one phenolic acid—glucosyringic acid—and three flavonoids—naringin, didimin (acinoside, isosakuranetin-7-*O*-rutinoside) and maesopsin-6-*O*-glucopyranoside—in the samples. These rare compounds were not detected in our research.

Under the present study, we confirmed that fruits are a poorer source of phenols than stems and leaves of *S. rubriflora* (Table 2, Table 3 and Table 4). Total phenolic content based on HPLC-DAD results was 431 mg/100 g DW for the fruit extracts (Table 2), and was 3–8-fold lower than the minimal and maximal contents detected for the investigated leaf and stem extracts. Total phenolic content ranged from 1233 (M, autumn) to 3283 mg/100 g DW (F, spring) (Table 3) for the stem extracts, and from 1689 (M, autumn) to 3452 mg/100 g DW (M, spring) for the leaf extracts (Table 4). The results of the chromatographic quantification fully correlated with the results of the total phenolic content assay carried out spectrophotometrically according to the Folin-Ciocaletu assay (Table 6). The highest total phenolic content was indicated for the extracts of leaves collected in spring (F—36,633 mg/100 g DW; M—31,207 mg/100 g DW), and was 7- and 6-fold lower for the fruit extracts (5181 mg/100 g DW) (Table 6). In the stem extracts, total phenolic content was marginally lower in comparison to leaves—22,764 mg/100 g DW for F and 18,129 mg/100 g DW for M specimens (Table 6).

Neochlorogenic acid, indicated as the most abundant compound in the stem and leaf extracts, was not detected in the fruit extracts. The high amount was confirmed for chlorogenic acid (69 mg/100 g DW) and kaempferol (138 mg/100 g DW) (Table 2). Fruits of *S. chinensis*, were investigated by Mocan et al. [23]. This team studied the material supplied by a local producer from Cluj-Napoca (Romania). It was confirmed with HPLC-UV-MS that fruits are a poor source of phenolic compounds. From among phenolic acids, only chlorogenic acid was detected in the amount of 0.33 mg/100 g DW and traces of genistic acid and *p*-coumaric acid were also found. The amount of chlorogenic acid was 27-fold lower than that detected in our fruit samples. Mocan et al. confirmed the presence of four compounds from among flavonoids, namely rutoside (1.3 mg/100 g DW), isoquercitrin (0.66 mg/100 g DW), hyperoside (0.2 mg/100 g DW), and quercetin (0.2 mg/100 g DW). These amounts were many times lower than that detected by us for *S. rubriflora*—they were 32-, 41-, 24-, and 180-fold lower, respectively. Further, these studies did not detect guaijaverin and kaempferol, which were the main compounds confirmed in the fruit extracts of *S. rubriflora* (Table 2). The differences in fruit extract phenolic composition between *S. rubriflora* and *S. chinensis* were also confirmed by our team. Using HPLC-DAD, we were able to confirm different compounds from among phenolic acids, including gallic acid, p-hydroxybenzoic acid, protocatechuic acid, salicylic acid, syringic acid, and vanillic acid, in *S. chinensis* fruits of Polish origin. Only chlorogenic acid was confirmed in fruits of both species, but in *S. chinensis* its amount was 2-fold lower (4.55 mg/100 g DW) than in *S. rubriflora*. Our former studies did not confirm the presence of flavonoid compounds in *S. chinensis* fruits [30,53,54,55,56].

*S. rubriflora* stem extracts proved to be abundant in phenolic compounds (Table 3). Their individual and total amounts were dependent on the harvesting time, vegetation period and the sex of the studied *S. rubriflora* individuals. Total phenolic content was very high, and in each vegetation period, higher content was found for stems collected from female specimens. Spring: F—3283 mg/100 g DW; M—2266 mg/100 g DW. Summer: F—2213 mg/100 g DW; M—1510 mg/100 g DW. Autumn: F—1756 mg/100 g DW; M—1233 mg/100 g DW (Table 3). The highest total phenolic content was found in the extracts of stems harvested in spring from F specimens, both for individual compounds and for the total pools. Neochlorogenic acid was clearly the main compound (max. 457 mg/100 g DW, F, spring) among the detected phenolic acids. Rutoside (max. 932 mg/100 g DW), isoquercitrin (624 mg/100 g DW) and trifolin (605 mg/100 g DW) were the quantitative dominant compounds among flavonoids. Their maximal content was confirmed in stems harvested in spring from F specimens. The stem extracts of *S. chinensis* of Romanian origin were studied by Mocan et al. [24]. In their research, identification of compounds was performed using LC-DAD-ESI-ToF-MS, while quantification was carried out using HPLC-DAD. From among phenolic acids, similarly to *S. rubriflora*, compounds from depside group were indicated in the extracts. The 3-*O*-p-coumarylquinic acid (63 mg/100 g DW) and neochlorogenic acid (37 mg/100 g DW) were the dominant compounds. Chlorogenic acid (24 mg/100 g DW) and 4-*O*-*p*-coumarylquinic acid (17 mg/100 g DW) were also estimated. From among flavonoids in *S. chinensis* stem extracts, Mocan et al. estimated mainly 7-*O*-rhamnosides—quercetin-3-*O*-glucoside-7-*O*-rhamnoside (78 mg/100 g DW) and kaempferol-3-*O*-glucoside-7-*O*-rhamnoside (77 mg/100 g DW)—as well as 3-*O*-glucosides—quercetin-3-*O*-glucoside (59 mg/100 g DW) and kempferol-3-*O*-glucoside (37 mg/100 g DW) [24].

*S. rubriflora* leaf extracts were the most abundant in phenolic compounds, which was confirmed by both chromatographic and spectrophotometric analyses (Table 4 and Table 6). HPLC-DAD estimations indicated that the individual as well as the total amounts of phenolic acids and flavonoids were dependent on the harvesting vegetation period and on the sex of the studied *S. rubriflora* individuals. Total phenolic content was very high, and the highest content was found for leaves collected in spring: F—2814 mg/100 g DW; M—3452 mg/100 g DW. In the samples collected in summer, these values were also very high: F—2145 mg/100 g DW; M—2165 mg/100 g DW, respectively. Leaves collected in autumn were a good source of phenolic compounds: F—2718 mg/100 g DW; M—1689 mg/100 g DW (Table 4). Neochlorogenic acid was the main compound (607 mg/100 g DW, F, autumn; 529 mg/100 g DW, F, spring; and 530 mg/100 g DW, M, spring) among the detected phenolic acids. Among flavonoids, the maximal content of rutoside (max. 841 mg/100 g DW), trifolin (714 mg/100 g DW), isoquercitrin (499 mg/100 g DW), and guaijaverin (269 mg/100 g DW) was found in leaves harvested in spring from M specimens. The maximal content—164 and 59 mg/100 g DW, respectively—of hyperoside and isorhamnetin was detected in extracts from M leaves collected in autumn. The leaf extracts of *S. chinensis* of Romanian origin were studied for phenolic acids and flavonoids by Mocan et al. as well [24]. The contents of these phenolic compounds in the leaf extracts were higher than in stems or fruits. This team also found more compounds quantitatively. Among phenolic acids, the dominant compounds were chlorogenic acid (499 mg/100 g DW), neochlorogenic acid (295 mg/100 g DW), trans-5-*O*-*p*-coumaroylquinic acid (105 mg/100 g DW), and protocatechuic acid (53 mg/100 g DW). The quantitatively dominant compounds among flavonoids in *S. chinensis* leaf extracts were kaempferol 3-*O*-glucoside-7-*O*-rhamnoside (568 mg/100 g DW), kaempferol 3-*O*-glucoside (512 mg/100 g DW) and quercetin 3-*O*-galactoside (249 mg/100 g DW). As part of our earlier research on a *S. chinensis* specimen of Polish origin, the phenolic acid and flavonoid contents in the leaf extracts were also determined using HPLC-DAD [30,53,54,55]. They were quantitatively similar to those found by Mocan et al. Chlorogenic acid (48 mg/100 g DW) and protocatechuic acid (25 mg/100 g DW) were the dominant compounds among phenolic acids, while quercitrin (100 mg/100 g DW), quercetin (70 mg/100 g DW) and myricetin (42 mg/100 g DW) were detected among flavonoids [30,53,54,55,56]. The high contents of chlorogenic acid (max. 635 mg/100 g DW) and rutoside (103 mg/100 g DW) were confirmed also in the leaf extracts of *Lycium barbarum* and *Lycium chinense* cultivated in Italy [57]. In this study, the qualitative and quantitative differences between *Lycium* species were noted—cryptochlorogenic acid was found only in *L. barbarum*, while quercetin-3-*O*-rutinoside-7-*O*-glucoside and quercetin-3-*O*-sophoroside-7-*O*-rhamnoside were found only in *L. chinense* leaves.

The initiation of *S. rubriflora* microshoot cultures and testing of the accumulation of the phenolic content in their biomass performed in this study aimed at assessing their usefulness in relation to the material derived from the field conditions. The results of this research turned out to be ultimately innovative. According to the literature review, there are no papers on *S. rubriflora* in vitro cultures. The results obtained prove to be very interesting from a cognitive and practical point of view. The two lines (F and M) of in vitro microshoot cultures were successfully initiated from leaf buds. In vitro microshoot cultures grew well on MS medium supplemented with 1 mg/L BA and 1 mg/L IBA as the plant growth regulators. Biomass growth was satisfactory; the estimated growth index was approximately three for a 30 day growth period. That was similar to *S. chinensis* in vitro cultures cultured in our laboratory before [30,55,56]. Total phenolic content detected by HPLC-DAD (Table 5) was 515 mg/100 g DW for F and 631 mg/100 g DW for M lines. These results corresponded to the outcomes achieved by the spectrophotometric assay with Folin-Ciocaletu reagent, which indicated that total phenolic content was 4729 mg/100 g DW for F and 6374 mg/100 g DW for M lines (Table 6). In accordance with this method, total phenolic content of in vitro microshoots was slightly lower for F lines and marginally higher for M lines than for fruits of mother plants (5181 mg/100 g DW) (Table 6). In comparison to the stem and leaf extracts, total phenolic content was 4.8- and 2.8-fold and 7.7- and 4.9-fold lower for F and M microshoot lines, respectively. Based on the chromatographic estimations carried out with UHPLC-DAD-ESI-MS^3^, the qualitative profile of phenolic compounds was the same for in vitro cultures as well as for the intact plant material (Table 1). It was evident from the quantitative analysis carried out using HPLC-DAD that neochlorogenic acid and cryptochlorogenic acid were the dominant compounds among phenolic acids: 81 and 103 mg/100 g DW, and 87 and 91 mg/100 g DW for F and M lines, respectively (Table 5). In in vitro culture extracts, aglycones were the quantitative dominant compounds among flavonoids: kaempferol (F—72 mg/100 g DW; M—91 mg/100 g DW), isorhamnetin (F—72 mg/100 g DW; M—80 mg/100 g DW) and quercetin (F—56 mg/100 g DW and M—87 mg/100 g DW) (Table 5). In order to carry out the comparative assessment of the results from *S. rubriflora* in vitro cultures, our previous long-term studies on *S. chinensis* in vitro cultures were taken into consideration [30,53,54,55,56]. In these studies on *S. chinensis* agar microshoot cultures, chlorogenic acid (max. 13 mg/100 g DW) and protocatechuic acid (max. 36 mg/100 g DW) were the main compounds from phenolic acid group, similarly to *S. rubriflora* [30,53,54,55,56]. The main compound from among flavonoids was quercitrin (max. 27 mg/100 g DW), which was not estimated in the *S. rubriflora* microshoots.

To sum up the phenolic content estimations, it is important to state that in plant material (fruits, stems and leaves) of *S. rubriflora* and *S. chinensis*, it is *S. rubriflora* that is a richer source of compounds with antioxidant potential. Fruits of both species are a poor source of both phenolic acids and flavonoids. However, the leaf and stem extracts seem to be promising in this respect. The noticeably high contents of these compounds were noted using chromatographic and spectrophotometric tests in the leaf extracts collected in spring. Furthermore, stems collected at this time of the year also indicated good results.

The contents of these compounds in extracts from in vitro cultures were lower than in the leaf or stem extracts. However, they were comparable to the results obtained for fruits which are *Schisandra* pharmacopeial raw material. The outcome of our research resulted in carrying out the comparative analyses using four assays, namely FRAP, DPPH, CUPRAC, and QUENCHER-CUPRAC, for extracts from leaves, stems and fruits, as well as also evaluating our biotechnological research using in vitro cultures.

The antioxidant potential estimations revealed that *S. rubriflora* has very high antioxidant potential (Table 7). The highest antioxidant power was indicated by means of all the applied methods for the leaf extracts. For the leaf extracts, the highest antioxidant capability was found for M specimens when using the FARP and CUPRAC assays; the results for F and M specimens were almost the same using the DPPH and the QENCHER-CUPRAC assays. High potential was also demonstrated for the stem extracts. The antioxidant capacity of in vitro cultured microshoots measured with the DPPH and the QENCHER-CUPRAC assays was comparable to the power of the fruit extracts (Table 7).

Results from the antioxidant activity assays fully corresponded with the results from the quantification of phenols described above.

Many authors claim that phenolic secondary metabolites, especially from among phenolic acids and flavonoids, are primarily responsible for the antioxidant capacity of different plant raw materials [3,4,58,59,60]. Lignans (especially dibenzocyclooctadiene lignans) are the most characteristic and the most frequently marked compounds for the *Schisandra* genus [12,19,32]. There are only a few publications on the analysis of phenolic compounds and lignans in *Schisandra* species, which mainly concern *S. chinensis* species. Mocan et al. [24] characterised the contribution of the single constituents, namely lignans and further phenolic compounds, to the overall antioxidant activity. The results of the complex study contributed to similar conclusions of our research. The team measured the antioxidant activity of leaves, stems and fruits using the trolox equivalent antioxidant capacity assay of different extracts against the stable synthetic ABTS•+ radical cation. In general, not lignans, but chlorogenic acid isomers and quercetin glycosides contributed over 80% of the total antioxidant activity for *S. chinensis*. [24].

Choi et al. [29] assessed the antioxidant activity of lignans using the 2′,7′-dichlorodihydrofluorescein diacetate (DCFH-DA) cellular-based analysis. The structure–activity relationships of various dibenzocyclooctadiene lignans exhibited that the exocyclic methylene functionality, which occurs only in individual structures of lignans, was essential for the antioxidant activity, with the benzoyloxy group probably enhancing such effects. Only one structure—schisandrene—was indicated as a responsible lignan. Zhang et al. [26] estimated the antioxidant and antiproliferative activities of five compounds from among lignans and terpenoids (d-epigalbacin, machilin G, chicanine, anwulignan, and epi-anwuweizic acid) and the extract from *S. chinensis* fruits. The antioxidant capacity was evaluated by the DPPH radical-scavenging assay. The result of DPPH measures indicated that *S. chinensis* crude fruit extract exhibited 7-fold higher activity (IC_50_ = 188) than the individual tested compounds (max. chicanine IC_50_ = 26). This is a premise that lignans and terpenoids are less responsible for *Schisandra* antioxidant activity. The close influence of polyphenolic compounds on the antioxidant activity was also indicated by other authors for *Thymus vulgaris, Salvia officinalis* and *Origanum majorana* [61], *Moringa oleifera* [62], *Rhus coriaria* [63], or *Malus* sp. [64], *Juglans* sp. [65], and *Quercus* sp. [66]. The results of our research based on the determination of phenolic compounds fully correlated with the antioxidant activity studies carried out by our team as well.

The antioxidant activity for in vitro cultures is also often compared to in vivo material [9]. Hakkim et al. [67] conducted a comparative study of the chemical composition and antioxidant property of *Ocimum sanctum* leaves, stems, and inflorescences and their in vitro callus cultures. The callus cultures were maintained on MS medium with 1 mg/L of 2,4-dichlorophenoxyacetic acid (2,4-D) with different concentrations of kinetin (0.1–0.5 mg/L). The distribution of phenolic compounds in these extracts was analysed using HPLC-PDA. In the intact plant extracts, more compounds were detected than in the callus extracts. These included isothymusin, ursolic acid, carnosic acid, eugenol, sinapic acid, and rosmarinic acid. However, rosmarinic acid was found to be the predominant phenolic acid in the callus extracts, with an amount (ca. 220 mg/100 g DW) that was 11-fold higher than in the studied leaves, stems, and inflorescences (ca. 20 mg/100 g DW). In this study, the antioxidant activity of the extracts was evaluated, among others, by means of the DPPH assay. The IC_50_ (expressed in mg of extract/mL) of the callus cultures was ca. 0.4 and was higher—ca. 0.6—for the intact plant parts.

Costa et al. performed a comparative study of in vitro and in vivo material of *Thymus lotocephalus* [68] and *Lavandula viridis* [69]. The study compared phenolic metabolites using HPLC-DAD and also antioxidant activities using the oxygen radical absorbance capacity (ORAC) assay. The main phenols in *T. lotocephalus* were phenolic acids—caffeic acid and rosmarinic acid—and flavones—luteolin and apigenin. In vitro cultures accumulated the large amounts of rosmarinic acid. The research indicated that in vitro cultures of *T. lotocephalus* showed slightly lower activity than a wild plant expressed with the ORAC assay—1.30 and 1.19 (mol trolox/g of extract) for the herb and callus cultures extracts, respectively. HPLC-DAD analyses for *L. viridis* showed that the dominant phenols were; from phenolic acids: 3-*O*-caffeoylquinic, 4-*O*-caffeoylquinic, 5-*O*-caffeoylquinic, and rosmarinic acids, and from flavonoids luteolin and pinocembrin. The water/ethanol extract from in vitro cultures contained the highest amount of the identified phenolics (51,653 mg/kg). The antioxidant tests showed that extracts from *L. viridis* (both wild plants and in vitro cultures) showed the ability to chelate Fe^2+^, scavenge free radicals and protect against lipid peroxidation.

Kuhlmann and Röhl [70] compared the phenolic content using HPLC-DAD and the antioxidant capacity with DPPH of different in vitro culture types, namely shoot culture, callus culture and cell suspension of *Rosmarinus officinalis.* The dominant compounds found were diterpenes (carnosic acid and carnosol) and depside (rosmarinic acid). The level of carnosic acid in the suspension culture was 3-fold lower than for the callus culture. The amounts of rosmarinic acid produced in the shoot and callus cultures were similar, whereas a higher amount of rosmarinic acid was measured in the suspension culture than in the shoot and callus cultures. The results of the DPPH radical-scavenging activity of the extracts showed that this activity was dependent in particular on the amount of rosmarinic acid.

Taveira et al. [71] also made a comparison of the phenolic composition only of in vitro material from shoot, callus and root cultures of *Brassica oleracea* var. *costata* and its antioxidant capacity using DPPH. The determination of phenolic compounds was carried out by HPLC-DAD. No phenolic compounds were identified in callus and root cultures. The presence of 36 compounds, which included flavonoids (kaempferol and quercetin derivatives), hydroxycinnamic acids, and hydroxycinnamic acyl glycosides, was confirmed in shoots. MS liquid medium with 0.1 mg/L NAA (1-naphthaleneacetic acid) and 2 mg/L BA was the best condition to produce the shoot culture biomass with the highest phenolic content and the antioxidant potential. The authors indicated that the phenolic content was responsible for the antioxidant power of *B. oleracea* var. *costata* in vitro shoots.

Królicka et al. [72] evaluated the antioxidant activity as well as the secondary metabolites of *Drosera aliciae* shoot cultures grown in vitro. The methanol extract from *D. aliciae* proved to be an effective antioxidant in both the DPPH and the FRAP assays. The antiradical potential was dependent on the estimated flavonoid contents.

To conclude, the biotechnological research results confirmed our observations regarding in vitro cultures of *S. rubriflora*. The accumulation of phenolic compounds in in vitro cultured biomass was lower in comparison to the field-grown plants parts, which is connected with its antioxidant potential. However, *S. rubriflora* in vitro cultured biomass could be an alternative, valuable source of natural antioxidants and an efficient tool for in vitro biosynthesis of phenolic acids (neochlorogenic and cryptochlorogenic acids) as well as for flavonoids (kaempferol, isorhamnetin), which would allow for avoiding the need to exploit populations of wild, endemic plants.

## 5. Conclusions

The present study is the first comparative, complex, qualitative and quantitative analysis of the polyphenol composition as well as of the antioxidant potential of *S. rubriflora* fruits, stems and leaves and in vitro microshoot cultures. The qualitative estimations of phenolic acids and flavonoids were performed using UHPLC-DAD-ESI-MS^3^. The phenolic profile revealed the presence of 27 compounds. The contents of the main compounds have been determined for the first time using HPLC-DAD. This study quantitatively characterised from phenolic acids: chlorogenic acid, cryptochlorogenic acid and neochlorogenic acid, from flavonoid glycosides: hyperoside, rutoside, isoquercitrin, guaijaverin, and trifolin, and from flavonoid aglycones: quercetin, kaempferol and isorhamnetin. The qualitative (fruits—no presence of neochlorogenic acid) and quantitative differences in the phenolic compound composition were recorded and they were dependent on the sex of specimens, the vegetation period, and parts of the intact plant material—fruits, leaves and stems. The qualitative and quantitative differences were also indicated for the first time in in vitro cultures of *S. rubriflora* initiated within this study.

Additionally, the antioxidant activity, based on four in vitro assays (DPPH, FRAP, CUPRAC and QUENCHER-CUPRAC), of *S. rubriflora* samples with the highest content of phenolic compounds (stems and leaves collected in spring), fruits and in vitro microshoots has been determined for the first time. The results of the antioxidant assays agreed with the results of total phenolic content measured spectroscopically with the Folin–Ciocalteu assay as well as with the chromatographic analyses. A close relationship between total phenolic content in the studied materials of *S. rubriflora* and their antioxidant potential has been documented for the first time. Moreover, the results revealed the high competitiveness of *S. rubriflora* in relation to the known, pharmacopeial plant species—*S. chinensis*.

As a result of our research, the extracts of *S. rubriflora* (fruit, stems and leaves) should be considered as a rich, valuable source of phenolic compounds, with promising very strong antioxidant potential. In vitro cultures exhibited very interesting differences and showed new research directions involving plant biotechnology solutions for obtaining this endemic plant material.

## Figures and Tables

**Table 1:** The phenolic profile of *S. rubriflora* extracts determined by UHPLC-DAD-ESI-MS^3^.

No. (See Figure 2)	Compound	Retention Time [min]	UV-Vis	Parent Ion [M − H]^−^ (*m/z*)	Daughter MS^2^ Ions	Daughter MS^3^ Ions	Parent Ion [M − H]^±^ (*m/z*)	Daughter MS^2^ Ions	Daughter MS^3^ Ions	Neutral Loss
1	Neochlorogenic acid ^s^*	9.9	241, 296sh, 324	353	191b, 179, 135	-	355	163b**, 145, 135	-	-
2	p-Coumaroylquinic acid isomer	13.1	224, 299sh, 311	337	191, 173, 163b, 119	-	339	147b, 119	-	-
3	p-Coumaroylquinic acid isomer	14.0	225, 300sh, 311	337	191, 173, 163b, 119	-	339	147b, 119	-	-
4	Chlorogenic acid ^s^	16.8	223, 300sh, 325	353	265, 191b, 179	-	355	-	-	-
5	Cryptochlorogenic acid	18.8	225, 300sh, 324	353	244, 191, 179, 173b, 135	-	355	-	-	-
6	p-Coumaroylquinic acid isomer	22.6	225, 299sh, 311	337	191b, 173, 163	-	339	147b, 119	-	-
7	p-Coumaroylquinic acid isomer	27.2	224, 300sh, 310	337	191b, 163, 136	-	339	147b, 119	-	-
8	Quercetin *O*-rhamnohexoside	36.3	255, 263sh, 353	609	591, 343, 301b, 271, 179	-	611	**465**, 303b	345, 303b	146
9	Hyperoside ^s^	36.9	255, 262sh, 353	463	343, **301b**	271, 255, 179b, 151	465	**303b**	285, 274, 257b, 229	162
10	Rutoside ^s^	37.2	255, 260sh, 353	609	343, 301b, 271, 179	-	611	**465**, 303b	303b	146
11	Isoquercitrin ^s^	38.0	255, 261sh, 353	463	343, **301b**	271, 255, 179b	465	**303b**	285, 274, 257b, 229	162
12	Guaijaverin ^s^	39.6	256, 260sh, 353	433	**301b**	271, 255, 179b, 151, 107	435	**303b**	285, 257b, 247, 187, 153	132
13	Trifolin ^s^	40.8	overlapped	447	419, 327, 285b, 255, 151	-	449	**287b**	259, 231b	162
14	Avicularin ^s^	40.8	overlapped	433	343, 301b	-	435	**303b**	285, 257b, 247, 153, 135	132
15	Kaempferol *O*-rhamnohexoside	42.2	265, 344	593	357, 327, 285b, 255, 151	-	595	**449**, 287b	287b	146
16	Astragalin ^s^	43.1	265, 244	447	357, 327, 285b, 255, 151	-	449	**287b**	259, 213b	162
17	Isorhamnetin *O*-rhamnohexoside	43.7	254, 261sh, 352	623	357, 315b, 300, 271, 255	-	625	**479**, 317b	317b	146
18	Kaempferol *O*-pentoside	44.6	265, 350	417	327, 285b, 255	-	419	**287b**	213b	132
19	Quercetin *O*-acetyl-*O*-rhamnohexoside	45.2	255, 261sh, 354	651	609b, 301, 591	-	653	**465**, 303b	303b	-
20	Kaempferol *O*-pentoside	45.5	265, 349	417	327, 285b, 255	-	419	287b	243, 213b	132
21	Kaempferol *O*-acetyl-*O*-rhamnohexoside	50.8	263, 349	635	593, 575, 327, 285b, 257	-	637	**449**, 287b	287b	-
22	Isorhamnetin *O*-acetyl-*O*-rhamnohexoside	51.5	253, 264sh, 354	665	623, 605, 315b, 300, 271, 255	-	667	**479**, 317b	317b	-
23	Quercetin *O*-diacetyl-*O*-rhamnohexoside	52.9	254, 261sh, 353	693	651b, 609, 301	-	695	465, 303b	-	-
24	Quercetin ^s^	55.8	252, 262sh, 365	301	273, 257, 179b, 151, 107	-	303	285, 257b, 229	-	-
25	Kaempferol *O*-diacetyl-*O*-rhamnohexoside	57.3	264, 349	677	635, 593, 327, 285b	-	679	661, 449, 287b, 231	-	-
26	Kaempferol ^s^	65.8	265, 364	285	267, 257, 226, 171, 151b, 137	-	287	386, 369b, 337, 309	-	-
27	Isorhamnetin ^s^	67.0	253, 260sh, 353	315	299b, 285	-	317	302b, 285	-	-

^s^*—compared with standard substance, b**—most abundant ion in the fragmentation spectrum, in bold—ions subjected to MS^3^ fragmentati

**Table 2:** The content (mg/100 g DW ± SD) of main detected phenolic compounds of *S. rubriflora* fruit extracts (*n* = 5, *p* < 0.05).

Group of Compounds	Compound	Fruit Extract Content
Phenolic acids	Neochlorogenic acid	nd*
	Chlorogenic acid	9 ± 1
	Cryptochlorogenic acid	69 ± 9
	Total phenolic acid content	78 ± 10
Flavonoids	Hyperoside (quercetin 3-galactoside)	5 ± 1
	Rutoside (quercetin 3-rutinoside)	42 ± 5
	Isoquercitrin (quercetin 3-glucoside)	27 ± 3
	Guaijaverin (quercetin 3-arabinoside)	52 ± 5
	Trifolin (kaempferol-3-*O*-galactoside)	19 ± 3
	Quercetin	31 ± 4
	Kaempferol	138 ± 10
	Isorhamnetin	39 ± 3
	Total flavonoid content	353 ± 35
Total phenolic content		431 ± 45

nd*—not detected.

**Table 3:** The content (mg/100 g DW ± SD) of main detected phenolic compounds of *S. rubriflora* stem extracts (F—female; M—male) (*n* = 5, *p* < 0.05).

Group of Compounds	Compound	Stem Extract Content					
		Time of Plant Material Harvesting					
		Spring		Summer		Autumn	
		F	M	F	M	F	M
Phenolic acids	Neochlorogenic acid	457 ± 59	326 ± 16	302 ± 25	185 ± 8	229 ± 15	190 ± 9
	Chlorogenic acid	53 ± 1	50 ± 6	50 ± 2	31 ± 3	18 ± 2	27 ± 3
	Cryptochlorogenic acid	80 ± 5	66 ± 5	45 ± 5	44 ± 6	20 ± 1	49 ± 6
	Total phenolic acid content	590 ± 65	442 ± 26	392 ± 32	260 ± 17	267 ± 18	266 ± 17
Flavonoids	Hyperoside (quercetin 3-galactoside)	86 ± 8	109 ± 12	78 ± 4	77 ± 8	60 ± 5	130 ± 15
	Rutoside (quercetin 3-rutinoside)	932 ± 78	524 ± 20	573 ± 9	343 ± 33	471 ± 44	213 ± 26
	Isoquercitrin (quercetin 3-glucoside)	624 ± 59	341 ± 11	382 ± 35	239 ± 13	307 ± 18	160 ± 2
	Guaijaverin (quercetin 3-arabinoside)	145 ± 12	190 ± 20	103 ± 7	99 ± 8	93 ± 5	84 ± 8
	Trifolin (kaempferol-3-*O*-galactoside)	605 ± 14	428 ± 28	470 ± 26	298 ± 22	364 ± 35	189 ± 8
	Quercetin	135 ± 15	123 ± 7	111 ± 11	82 ± 6	88 ± 9	71 ± 7
	Kaempferol	113 ± 5	72 ± 2	84 ± 2	72 ± 6	72 ± 8	74 ± 6
	Isorhamnetin	53 ± 2	36 ± 4	45 ± 3	41 ± 5	33 ± 4	47 ± 4
	Total flavonoid content	2693 ± 193	1824 ± 104	1816 ± 96	1249 ± 101	1489 ± 128	967 ± 77
Total phenolic content		3283 ± 258	2266 ± 131	2213 ± 128	1510 ± 118	1756 ± 146	1233 ± 94

**Table 4:** The content (mg/100 g DW ± SD) of main detected phenolic compounds of *S. rubriflora* leaf extracts (F—female; M—male) (*n* = 5, *p* < 0.05).

Group of Compounds	Compound	Leaf Extract Content					
		Time of Plant Material Harvesting					
		Spring		Summer		Autumn	
		F	M	F	M	F	M
Phenolic acids	Neochlorogenic acid	529 ± 19	530 ± 17	217 ± 18	220 ± 23	607 ± 26	312 ± 18
	Chlorogenic acid	20 ± 1	34 ± 4	17 ± 1	25 ± 2	93 ± 9	35 ± 2
	Cryptochlorogenic acid	19 ± 1	50 ± 5	29 ± 3	53 ± 7	58 ± 3	94 ± 4
	Total phenolic acid content	568 ± 21	614 ± 26	262 ± 21	299 ± 32	758 ± 39	441 ± 23
Flavonoids	Hyperoside (quercetin 3-galactoside)	67 ± 3	145 ± 26	43 ± 4	106 ± 6	96 ± 6	164 ± 14
	Rutoside (quercetin 3-rutinoside)	798 ± 51	841 ± 62	596 ± 37	476 ± 17	594 ± 28	311 ± 23
	Isoquercitrin (quercetin 3-glucoside)	502 ± 47	499 ± 49	433 ± 28	306 ± 28	433 ± 20	180 ± 32
	Guaijaverin (quercetin 3-arabinoside)	92 ± 12	269 ± 39	95 ± 5	183 ± 8	83 ± 10	95 ± 5
	Trifolin (kaempferol-3-*O*-galactoside)	576 ± 42	714 ± 60	457 ± 34	511 ± 35	458 ± 50	251 ± 18
	Quercetin	111 ± 2	213 ± 11	116 ± 13	116 ± 8	129 ± 4	102 ± 7
	Kaempferol	66 ± 13	109 ± 8	95 ± 6	110 ± 12	111 ± 15	85 ± 5
	Isorhamnetin	33 ± 2	48 ± 6	49 ± 2	58 ± 7	59 ± 4	59 ± 4
	Total flavonoid content	2246 ± 172	2838 ± 261	1883 ± 128	1866 ± 122	1961 ± 138	1248 ± 108
Total phenolic content		2814 ± 194	3452 ± 287	2145 ± 149	2165 ± 154	2718 ± 177	1689 ± 131

**Table 5:** The content (mg/100 g DW ± SD) of main detected phenolic compounds of *S. rubriflora* microshoot in vitro culture extracts (F—female; M—male) (*n* = 10, *p* < 0.05).

Group of Compounds	Compound	Microshoot In Vitro Culture Extracts Content	
		F	M
Phenolic acids	Neochlorogenic acid	81 ± 6	103 ± 9
	Chlorogenic acid	20 ± 2	17 ± 1
	Cryptochlorogenic acid	87 ± 2	91 ± 3
	Total phenolic acid content	188 ± 10	211 ± 13
Flavonoids	Hyperoside (quercetin 3-galactoside)	14 ± 1	12 ± 1
	Rutoside (quercetin 3-rutinoside)	29 ± 1	42 ± 1
	Isoquercitrin (quercetin 3-glucoside)	21 ± 2	26 ± 2
	Guaijaverin (quercetin 3-arabinoside)	38 ± 1	44 ± 2
	Trifolin (kaempferol-3-*O*-galactoside)	27 ± 1	37 ± 4
	Quercetin	56 ± 2	87 ± 5
	Kaempferol	72 ± 3	91 ± 4
	Isorhamnetin	72 ± 5	80 ± 6
	Total flavonoid content	328 ± 16	420 ± 25
Total phenolic content		515 ± 27	631 ± 38

**Table 6:** Total phenolic content of plant material determined by Folin-Ciocaletu assay. Antioxidant response was expressed as TE in mg/100 g DW ± SD; F—female; M—male (*n* = 4, *p* < 0.05).

Sample		Total Phenolic Content
Plant material	Fruit	5181 ± 63
	Stem F	22,763 ± 679
	Stem M	18,129 ± 524
	Leaf F	36,633 ± 447
	Leaf M	31,207 ± 381
In vitro cultures	Microshoot F	4729 ± 58
	Microshoot M	6374 ± 78

**Table 7:** Antioxidant potential of plant material determined by Ferric-Reducing Antioxidant Power (FRAP), 2,2-Diphenyl-1-Picryl-Hydrazyl-Hydrate (DPPH), Cupric-Reducing Antioxidant Capacity (CUPRAC) and QUick, Easy, New, CHEap, and Reproducible CUPRAC (QUENCHER-CUPRAC) assays. Antioxidant response was expressed as TE in mg/100 g DW ± SD; F—female; M—male (*n* = 4, *p* < 0.05).

Sample		FRAP	DPPH	CUPRAC	QUENCHER-CUPRAC
Plant material	Fruit	10,075 ± 225	2984 ± 75	10,075 ± 358	6083 ± 757
	Stem F	7474 ± 962	7967 ± 124	22,270 ± 655	27,147 ± 692
	Stem M	5893 ± 135	8340 ± 163	17,920 ± 488	28,317 ± 696
	Leaf F	19,720 ± 440	8405 ± 210	19,720 ± 701	29,539 ± 1606
	Leaf M	28,179 ± 629	8363 ± 209	28,179 ± 1002	29,303 ± 1940
In vitro cultures	Microshoot F	2869 ± 64	2964 ± 74	2869 ± 102	5994 ± 556
	Microshoot M	5178 ± 116	3750 ± 94	5178 ± 184	5822 ± 579
